# Comparative evaluation of amiodarone and furosemide compatibility under different mixing conditions

**DOI:** 10.1038/s41598-025-20217-0

**Published:** 2025-10-17

**Authors:** So Iwabuchi, Toru Imai, Naoto Suzuki, Hiroshi Nango, Taiki Nagatomo, Hiroko Miyagishi, Toyofumi Suzuki, Susumu Ootsuka, Yasuhiro Kosuge

**Affiliations:** 1 School of Pharmacy Laboratory of Pharmacology , Nihon University , Chiba, Japan; 2 Department of Pharmacy , Nihon University Itabashi Hospital , Tokyo, Japan; 3 School of Pharmacy Laboratory of Pharmaceutics , Nihon University , Chiba, Japan; 4 Department of Pharmacy , Nihon University Hospital , Tokyo, Japan

**Keywords:** Amiodarone, Furosemide, Mixing, Physical compatibility, Intensive care unit, Medication safety, Diseases, Drug discovery, Medical research

## Abstract

Amiodarone and furosemide are frequently administered together in the intensive care unit, and their co-administration poses a risk of precipitation and catheter occlusion due to incompatibility. Thus, compatibility should be evaluated under clinically relevant conditions to ensure safe drug administration. This study investigated the impact of different mixing procedures on the physical compatibility of amiodarone and furosemide by employing two clinically relevant approaches: simulated mixing (SM) and route mixing (RM). Mixtures were prepared at therapeutic concentrations and evaluated by visual inspection, absorbance measurement, powder X-ray diffraction (PXRD), pH analysis, and particle size assessment. Although the drug ratios were identical, the RM samples displayed greater turbidity than the SM samples. The PXRD analysis showed no formation of new crystalline substances, and the precipitates consisted of known furosemide and amiodarone hydrochloride polymorphs. The pH shifted toward the alkaline range as the injection volume increased with furosemide dose. Importantly, differences in particle growth behavior were noted between SM and RM, with larger particles observed in the RM samples. In conclusion, mixing route significantly affects drug compatibility, with RM presenting a higher risk of precipitation. Assessing drug compatibility under clinically relevant conditions may help prevent catheter-related complications and improve the safety of intravenous pharmacotherapy.

## Introduction

Critically ill patients admitted to the intensive care unit (ICU) often require treatment for various underlying diseases and complications, with each patient receiving an average of six different medications^1^. In principle, each intravenous drug should be administered through a dedicated line to prevent incompatibility. However, in day-to-day practice, 47% of patients in the ICU receive three or more medications simultaneously via the same infusion route^2^. Furthermore, 8.5% of ICU cases involve the administration of drugs with known or unrecognized incompatibilities via the same route, hindering the safe implementation of pharmacological therapy^3^. Consequently, the risk of drug incompatibilities increases, prompting numerous studies to evaluate their compatibility with commonly used ICU medications^4,5^. Incompatibility with injectable drugs can lead to decreased efficacy and the risk of embolism due to insoluble precipitates, markedly affecting the prognosis of critically ill patients^6–8^. Therefore, verifying the compatibility of each drug is essential when administering multiple medications via the same route to avoid potential incompatibilities and ensure patient safety.

In the ICU, life-threatening arrhythmias such as ventricular fibrillation (VF) and ventricular tachycardia (VT) are associated with various conditions, including acute heart failure and myocardial infarction^9,10^. During such emergencies, multiple medications are promptly administered. Amiodarone is widely used as a first-line agent owing to its broad pharmacological effects against VF and VT^11–13^. Additionally, patients with VF or VT often present with circulatory failure, which may lead to pulmonary congestion and edema, making fluid management crucial for prognosis^14^. Therefore, furosemide is commonly co-administered with amiodarone. Amiodarone is poorly soluble in water but remains soluble under acidic conditions (approximately pH 4)^15^; therefore, its compatibility with drugs formulated under basic conditions must be evaluated. Furosemide, an acidic drug formulated at a basic pH of 8.6–9.6 to enhance its solubility, can precipitate when mixed with drugs formulated under acidic conditions^16^. To minimize drug incompatibility, furosemide should be administered either alone or with drugs formulated under basic conditions^17^. Nevertheless, despite the frequent co-administration of amiodarone and furosemide in clinical settings, reports on their compatibility are inconsistent, with some studies indicating compatibility^18^ and others reporting incompatibility^17^. These discrepancies could be attributed to differences in testing methods that do not accurately reflect clinical administration conditions. Therefore, compatibility must be re-evaluated under conditions that better simulate clinical settings.

Various methods have been employed to assess the physical compatibility of intravenous pharmaceutical formulations, including mixing drugs in a 1:1 ratio in a test tube^19^, varying the mixing ratio in the test tube^20^, and using intravenous infusion routes^21^. During intravenous administration, simultaneous administration of multiple drugs typically occurs via branching sites, such as Y-sites or three-way stopcocks. Intravenous administration from the Y site results in a 1:1 volume mixture^22^, leading to the common practice of performing compatibility tests at this ratio. However, differences in the mixing ratio cause variations in the incompatibility outcomes^23^. Multiple drugs are often administered through branching sites via infusion or syringe pumps in patients in the ICU. In these cases, the mixed fluids in the tubular structures of the infusion routes exhibit either laminar or turbulent flow behavior^24^, which may prevent uniform drug mixing. Furthermore, fluctuations in the flow rate of each drug caused constant changes in the mixing ratios. Consequently, the application of traditional compatibility testing methods in the clinical setting is challenging. These factors necessitate the development of compatibility testing methods that can better simulate clinical conditions. This study aimed to compare a conventional compatibility testing method with an approach that more closely simulates the clinical route of intravenous administration using amiodarone and furosemide as model compounds.

## Results

### Changes in appearance in each mixed solution for simulated mixing (SM) and route mixing (RM)

**Fig. 1:**
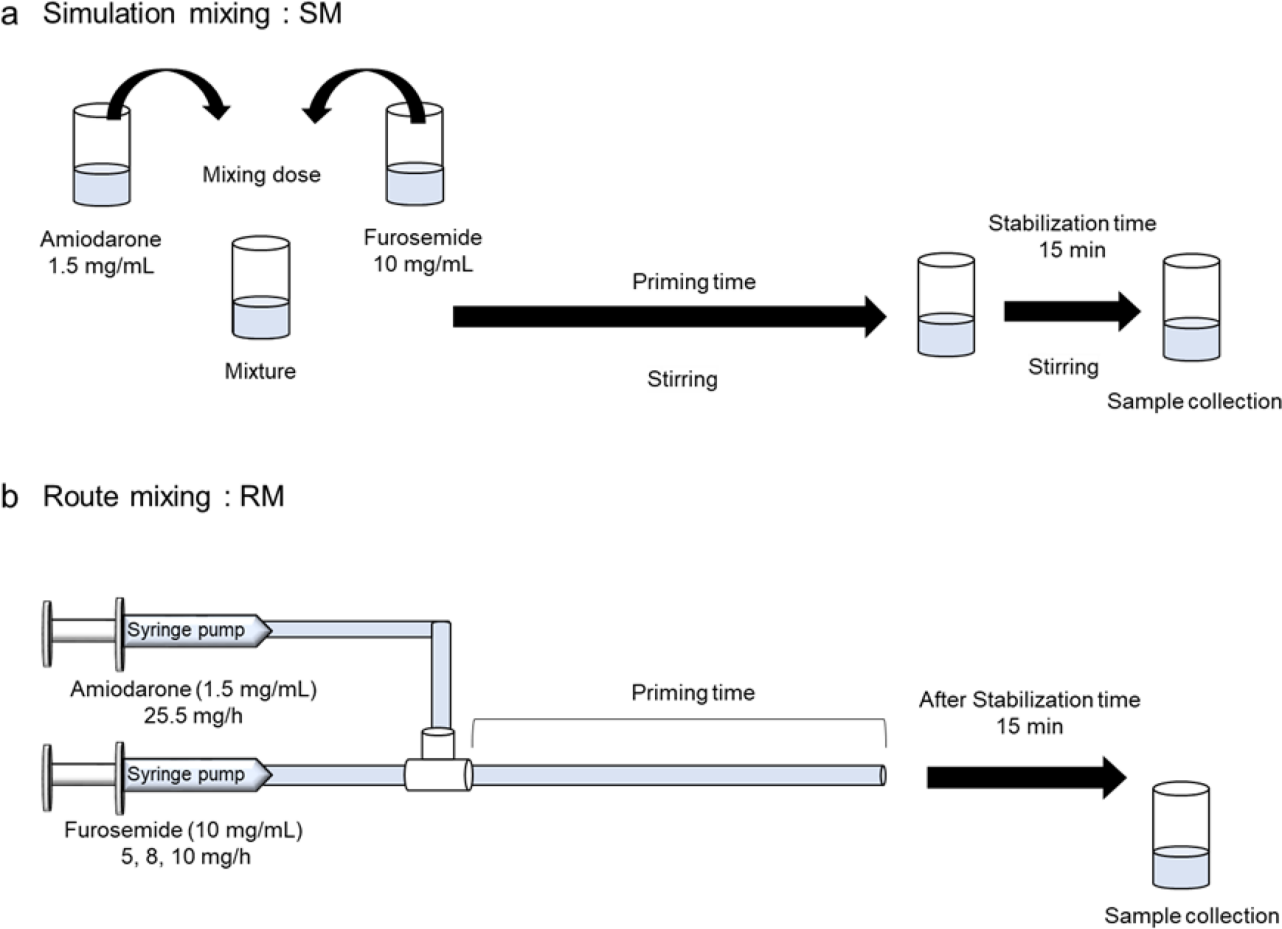
Method for preparing a mixture of amiodarone (adjusted to 1.5 mg/mL with 5% dextrose) and undiluted 10 mg/mL furosemide. (a) The two drug solutions were directly mixed in a screw-cap tube based on the dosage ratio used in clinical practice (simulation mixing [SM]). (b) The drug solutions were mixed under actual infusion conditions using a clinical infusion route at the clinically used dosage (route mixing [RM])

The optimal sampling time for the RM method was determined by monitoring visual changes at various time points after the infusion was initiated. No visible changes were observed immediately after the start of the furosemide infusion (8 or 10 mg/h). However, turbidity was noted in samples collected from the distal end of the infusion line 15 min after infusion initiation and persisted thereafter. In contrast, furosemide infusion at 5 mg/h showed no change, even after 90 min (data not shown). Therefore, for all subsequent experiments, RM samples were collected 15 min post-infusion. Similarly, for the SM samples, stirring was performed for a period equivalent to the RM priming time and continued for an additional 15 min (Fig. 1).

**Fig. 2:**
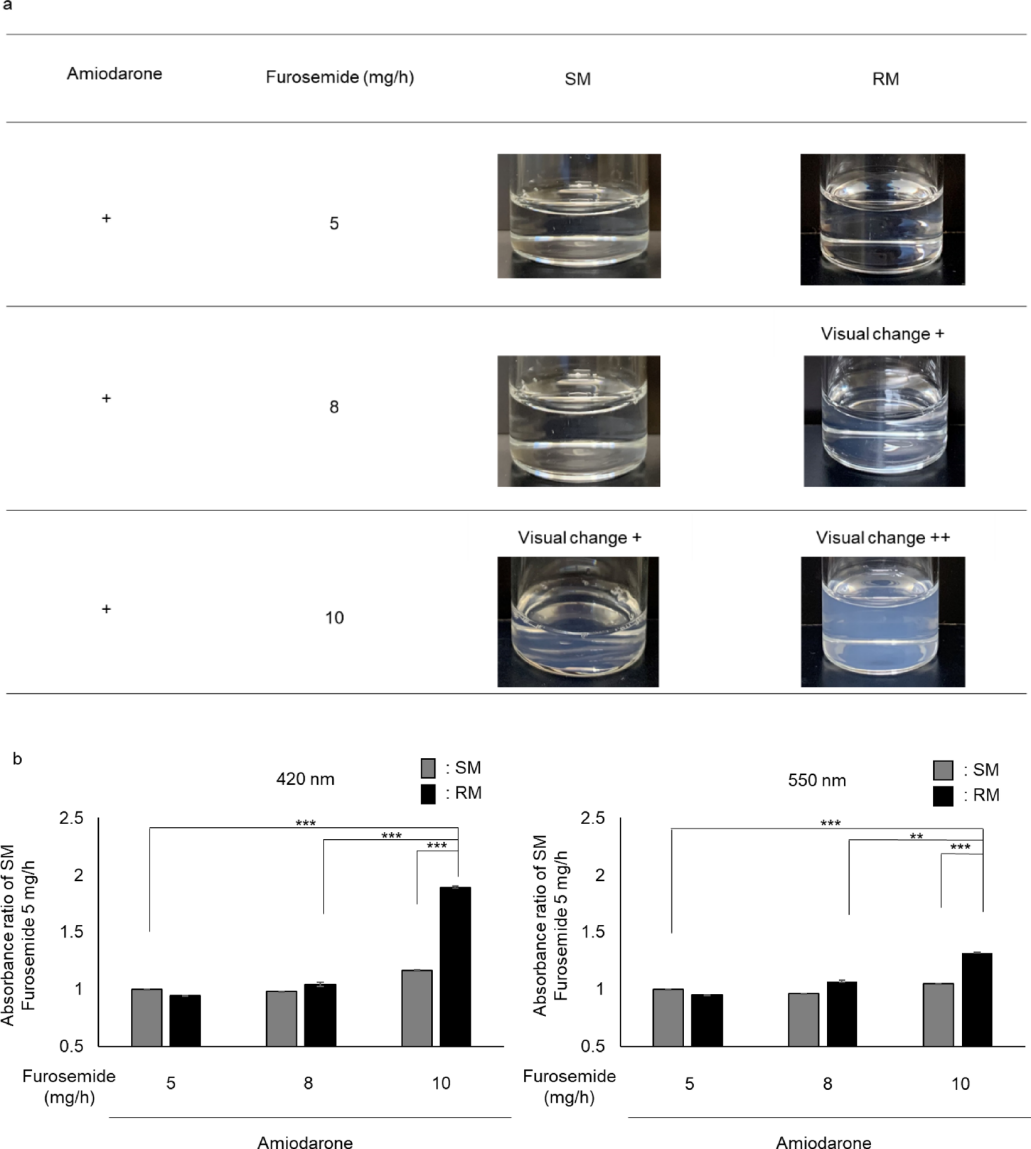
Visual changes and absorbance of amiodarone and furosemide mixtures prepared using the SM and RM methods. (a) Visual observation of the mixtures. Amiodarone was infused at 25.5 mg/h (corresponding injection volume: SM 1.7 mL, RM 17 mL/h). Furosemide was infused at 5 mg/h (SM 50 µL, RM 0.5 mL/h), 8 mg/h (SM 80 µL, RM 0.8 mL/h), or 10 mg/h (SM 100 µL, RM 1.0 mL/h) (b) Relative changes in absorbance at 420 nm and 550 nm in each group compared to those of amiodarone and furosemide 5 mg/h in SM. Data are presented as the mean ± standard error of three samples. ^**^ p < 0.01, ^***^ p < 0.001: Tukey’s multiple comparison test

Changes in the appearance of the mixed solutions prepared using the SM and RM methods were visually evaluated (Fig. 2a). No visible changes were observed in the amiodarone and furosemide mixtures at a rate of 5 mg/h under either condition. In contrast, faint turbidity was observed in the RM mixture with furosemide at 8 mg/h, whereas the SM mixture remained clear. Furthermore, turbidity was observed in both the SM and RM mixtures at a furosemide infusion rate of 10 mg/h, with more pronounced turbidity in the RM mixture than in the SM mixture.

Visible absorbance measurements were performed at 420 nm (indicative of color change) and 550 nm (indicative of turbidity) to assess changes in appearance quantitatively (Fig. 2b). The rate of change in absorbance at 420 nm showed no significant differences between SM mixtures at 8 and 10 mg/h (0.98 ± 0.01 and 1.17 ± 0.08, respectively) compared to 5 mg/h. Similarly, the absorbance values for RM mixtures at 5 and 8 mg/h (0.95 ± 0.03 and 1.04 ± 0.10, respectively) did not differ significantly from those of SM mixtures at 5 mg/h. However, RM mixtures at 10 mg/h showed a significantly higher absorbance (1.90 ± 0.10) than all other mixed solutions. Furthermore, the rate of change in absorbance at 550 nm showed no significant difference between SM mixtures at 8 and 10 mg/h (0.96 ± 0.01 and 1.05 ± 0.04, respectively) and SM mixtures at 5 mg/h. Similarly, no significant difference was observed between SM mixtures at 5 mg/h and RM mixtures at 5 and 8 mg/h (0.95 ± 0.03 and 1.01 ± 0.01, respectively). In contrast, RM mixtures at 10 mg/h exhibited a significant increase (1.37 ± 0.03) in turbidity compared to all other groups. The RM mixture with furosemide at 10 mg/h showed the most pronounced turbidity among all the conditions tested. The absorbance values were approximately 1.6 times higher at 420 nm and 1.3 times higher at 550 nm than the corresponding SM mixture. In contrast, the RM mixture at 8 mg/h exhibited only slight turbidity, and its absorbance did not differ significantly from that of the SM mixture at the same dose.

### Crystallinity analysis of white precipitates formed by the amiodarone and furosemide mixture

**Fig. 3:**
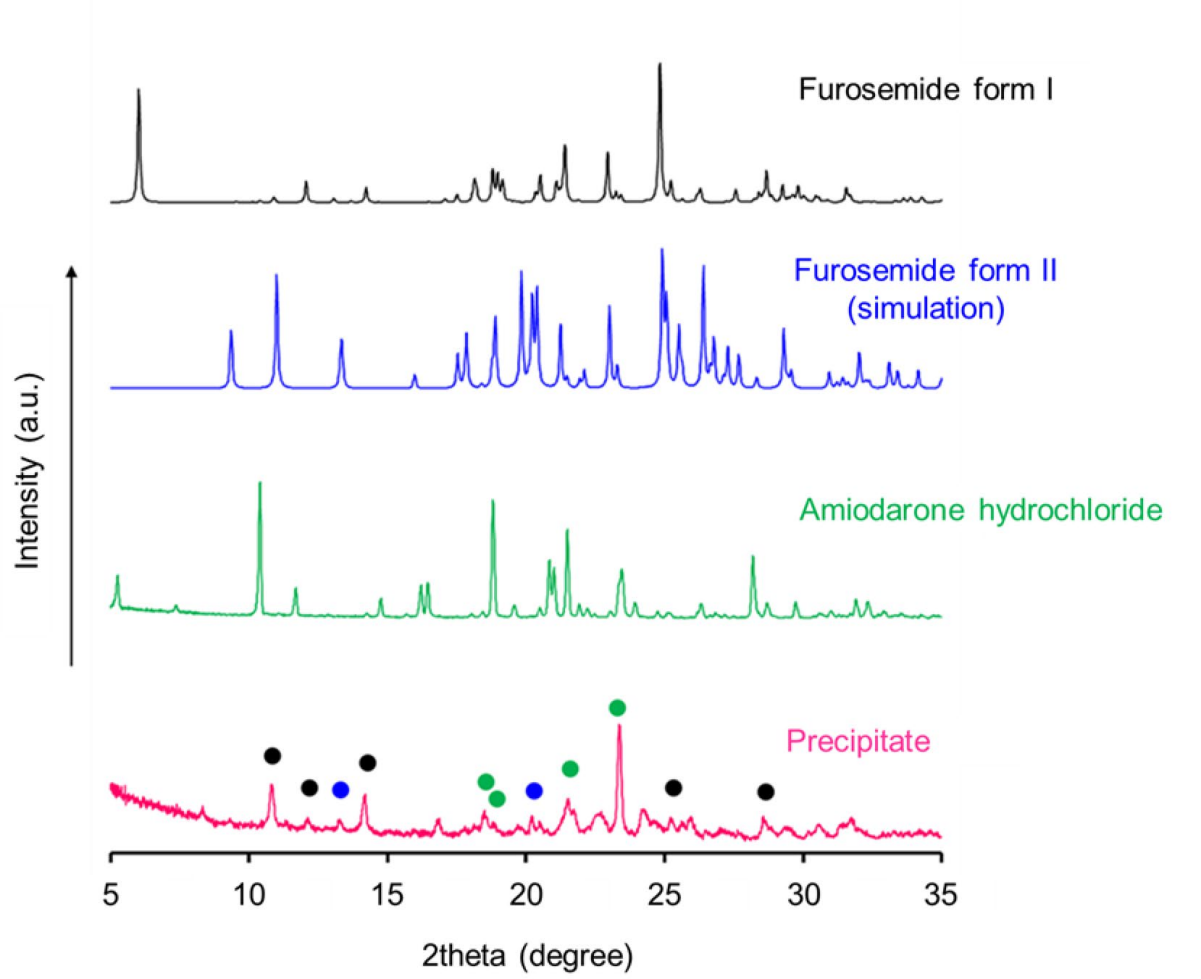
Powder X-ray diffraction patterns of white precipitates. Circular symbols indicate characteristic diffraction peaks derived from each crystalline component; black circles represent furosemide form I, blue circles represent furosemide form II, and green circles represent amiodarone hydrochloride

The crystallinity of the precipitates obtained from the turbid mixtures was evaluated using Powder X-ray diffraction (PXRD) to determine whether mixing amiodarone and furosemide formed a new structural entity. The PXRD pattern of the precipitate obtained after mixing exhibited diffraction peaks corresponding to furosemide and amiodarone hydrochloride (Fig. 3). Peaks attributable to furosemide forms I and II were also observed. Additionally, several diffraction peaks could not be assigned to the known crystalline forms of furosemide or amiodarone hydrochloride, suggesting a potential contribution from excipients in the formulations.

### Effect of SM and RM on pH

**Fig. 4:**
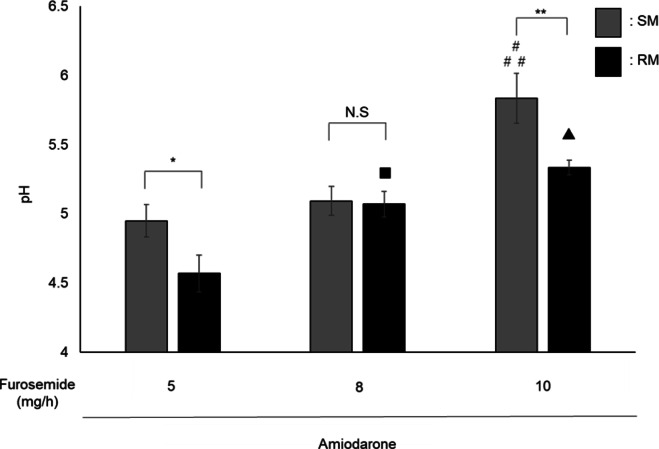
pH changes in mixtures containing varying amiodarone and furosemide doses The graphs illustrate the pH changes in the mixtures prepared using SM and RM containing different amiodarone and furosemide doses, along with the pH differences between the two methods. Amiodarone was infused at 25.5 mg/h (corresponding injection volume: SM 1.7 mL, RM 17 mL/h). Furosemide was infused at 5 mg/h (SM 50 µL, RM 0.5 mL/h), 8 mg/h (SM 80 µL, RM 0.8 mL/h), or 10 mg/h (SM 100 µL, RM 1.0 mL/h). Data are presented as the mean ± standard deviation of three samples. N.S. not significant, ^*^p< 0.05, ^**^p < 0.01, ^#^p < 0.001 SM 5 mg/h vs. SM 10 mg/h, ^##^p < 0.001 SM 8 mg/h vs. SM 10 mg/h, ^▲^p< 0.001 RM 5 mg/h vs. RM 10 mg/h, ^■^p < 0.05 RM 5 mg/h vs. RM 8 mg/h: Tukey’s multiple comparison test.

Mixing amiodarone and furosemide may result in formulation changes due to pH variations. Therefore, the pH of each mixed solution was determined. In the SM method, the pH values were 4.9 ± 0.1, 5.1 ± 0.1, and 5.8 ± 0.2 for furosemide doses of 5, 8, and 10 mg/h, respectively. In the RM method, the pH values were 4.6 ± 0.1, 5.1 ± 0.1, and 5.3 ± 0.1 for the corresponding doses. In both methods, the pH shifted toward more basic values as the injection volume increased with furosemide dose (Fig. 4).

The pH values for each mixing method were compared. In SM, the pH at 10 mg/h was significantly higher than that at 5 and 8 mg/h, whereas no significant difference was observed between 5 and 8 mg/h. A similar trend was noted for the RM method, where the pH at 8 and 10 mg/h was significantly higher than that at 5 mg/h; however, no significant difference was observed between 8 and 10 mg/h. When comparing SM and RM at the same furosemide dose, the pH in SM was significantly higher than that in RM at 5 and 10 mg/h, whereas no significant difference was observed between the two methods at 8 mg/h (Fig. 4).

### Effect of SM and RM on particle size

**Fig. 5:**
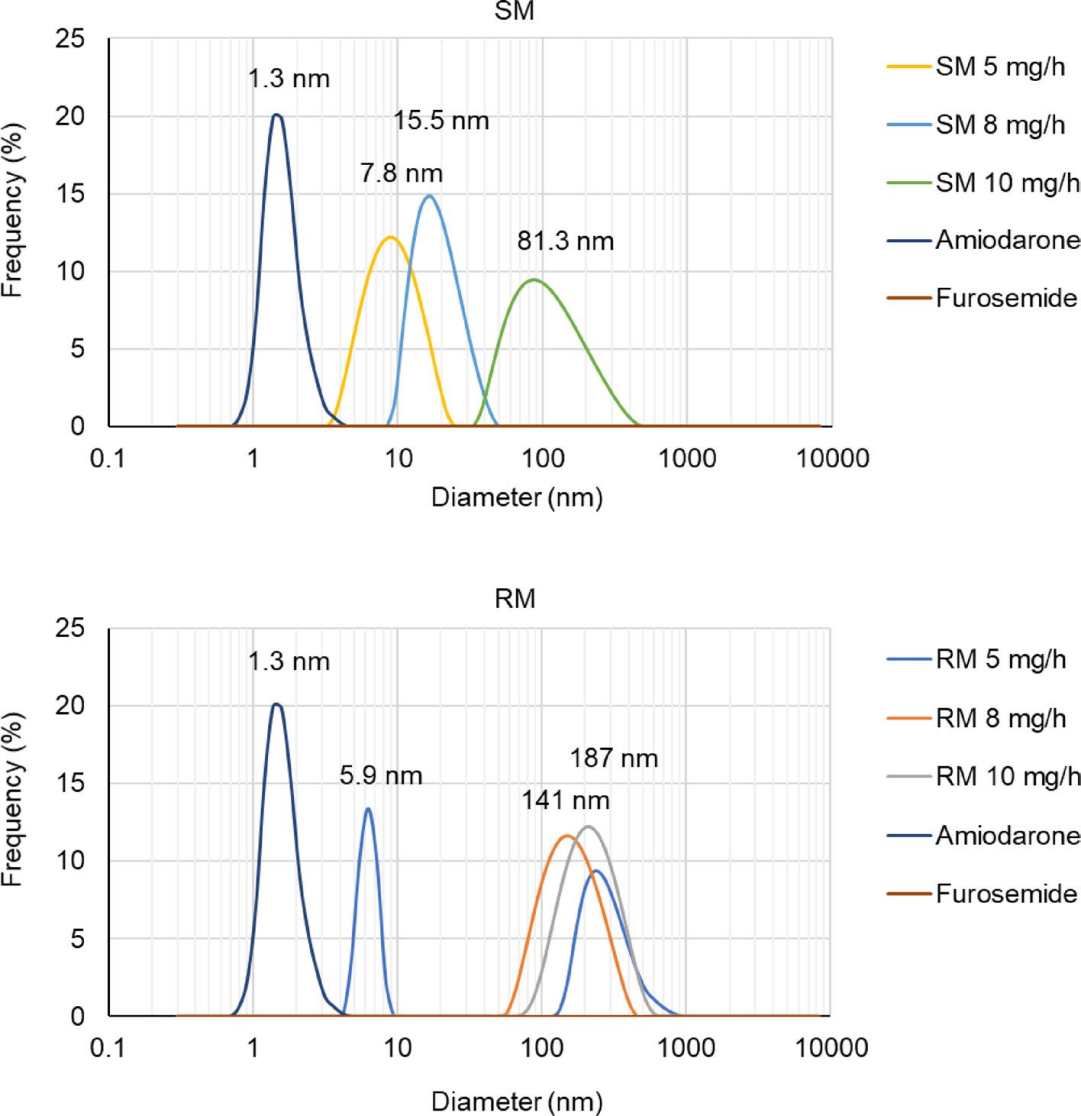
Particle size distribution analysis in amiodarone–furosemide mixtures. Comparative assessment of particle sizes in amiodarone and furosemide mixtures prepared using SM and RM methods, as measured by DLS. Amiodarone was infused at 25.5 mg/h (corresponding injection volume: SM 1.7 mL, RM 17 mL/h). Furosemide was infused at 5 mg/h (SM 50 µL, RM 0.5 mL/h), 8 mg/h (SM 80 µL, RM 0.8 mL/h), or 10 mg/h (SM 100 µL, RM 1.0 mL/h). The data illustrate the differences in the particle formation characteristics between the two preparation methods. Values are expressed as the median particle size for each mixture.

Dynamic light scattering (DLS) was used to measure the particle size distribution and assess how differences in appearance between the SM and RM methods affected the particle size. In the SM method, the mode diameter increased from 7.8 nm to 15.5 nm and 81.3 nm as the furosemide dose increased from 5 mg/h to 8 mg/h and 10 mg/h, respectively. In contrast, the RM method showed mode diameters of 5.9, 141, and 187 nm for the same doses, indicating a markedly different particle size behavior between the two methods. Additionally, a bimodal distribution was observed using the RM method at a furosemide dose of 5 mg/h, with particle populations of approximately 5.9 nm and 200 nm. The mode diameter of amiodarone alone at 1.5 mg/mL was 1.3 nm, whereas the particle size of furosemide at 10 mg/mL, which was dissolved entirely, could not be measured (Fig. 5).

## Discussion

Previous compatibility studies have primarily focused on mixing and stirring the two drugs under static conditions in test tubes. However, in clinical practice, drugs are administered continuously, mixed, and stirred via intravenous infusion routes and delivered into the body under dynamic conditions. This approach differs substantially from the static conditions typically used in test tube studies. Nonetheless, a direct comparison between the SM and RM methods has not been sufficiently investigated. This study revealed significant differences in drug incompatibility between the SM and RM methods for the combination of amiodarone and furosemide.

Several studies have investigated the compatibility of amiodarone and furosemide. For instance, no visual changes were observed when amiodarone (900 mg) was diluted in 500 mL of 5% glucose and mixed with furosemide (100 mg/10 mL)^18^. In contrast, visual changes occurred when higher concentrations of both drugs (15 mg/mL amiodarone and 10 mg/mL furosemide) were mixed at a 1:1 ratio^17^, suggesting that both the concentration and mixing ratio influence compatibility. In this study, the mixing ratios of amiodarone and furosemide were standardized using both the SM and RM methods, and the visual appearance and visible absorbance of the resulting mixtures were compared. When amiodarone was co-administered with furosemide at a rate of 5 mg/h, no visible changes were observed using either method. However, at 8 and 10 mg/h, apparent differences in visual changes were noted between SM and RM. Similarly, significant differences in visible absorbance were observed between SM and RM treated with furosemide at 10 mg/h. In contrast, no significant differences in the absorbance were detected at 8 mg/h; however, slight changes were observed. Visual detection of changes in appearance may be more sensitive than absorbance measurements^25^. Therefore, one possible explanation for the discrepancy between the visual changes and absorbance results is that absorbance measurements may have a lower sensitivity than visual assessments. In this study, both visual and absorbance measurements were used to assess the differences between SM and RM, demonstrating variations in compatibility. In other words, even with the same mixing ratio, differences in appearance compatibility were observed between SM and RM, emphasizing the influence of distinct mixing conditions on the results.

The mechanism underlying drug compatibility has been reported in several studies, with one example being the decrease in solubility of drugs, such as midazolam, owing to pH changes^26^ and the formation of insoluble salts upon mixing ceftriaxone and calcium preparations^27^. In this study, PXRD confirmed that the precipitate consisted of a mixture of furosemide polymorphs and amiodarone hydrochloride, without the formation of new crystalline substances. This finding suggests that the solubility of the drugs may have decreased upon mixing, leading to the precipitation of existing crystalline components. Furosemide exhibits pH-dependent solubility^28^, whereas the solubility of amiodarone hydrochloride is affected by temperature and pH^29,30^. In this study, all experiments were conducted under controlled room temperature conditions (20–25 °C), and the effect of temperature on the differences in turbidity between SM and RM was likely minimal. Therefore, the incompatibility between amiodarone and furosemide is primarily due to the decrease in solubility induced by changes in pH. This phenomenon can be explained by the Henderson–Hasselbalch equation and the ionization states of the drugs. Amiodarone is a basic drug (pKa 8.9) that is soluble in its protonated, ionized form under acidic conditions. Conversely, furosemide is an acidic drug (pKa 3.9) that is soluble in its deprotonated, ionized form under basic conditions. When the two solutions are mixed, the pH shifts to an intermediate range (4.5–6.0) in which a substantial fraction of both drugs exists in their less soluble, non-ionized forms, leading to precipitation.

The pH of each mixed solution was measured using SM and RM to investigate the effect of pH on the incompatibility between amiodarone and furosemide. In both methods, increasing the furosemide dose raised the injection volume, shifted the pH of the mixture toward the basic range, and caused visible changes in appearance. Generally, furosemide is highly stable under basic conditions, with a pH near 8.0. Specifically, its solubility is 0.33 mg/mL at pH 5.0 and 1.50 mg/mL at pH 6.5^31^. The furosemide doses used in this study, 5, 8, and 10 mg/h, correspond to calculated concentrations of 0.28, 0.45, and 0.55 mg/mL, respectively, all of which fall within the solubility range of pH 5–6 (0.33–1.50 mg/mL). In contrast, amiodarone exhibits the highest solubility at an acidic pH of approximately 4.5^32^, and its solubility decreases drastically between pH 5 and 6^33^. Therefore, an increase in pH is unlikely to cause furosemide precipitation, and the reduced solubility of amiodarone is likely the primary cause of turbidity.

Notably, under the furosemide 8 mg/h condition, turbidity was observed only in RM, despite no significant pH difference between SM and RM. This result suggests that turbidity cannot be explained solely by the pH-induced precipitation of amiodarone. Amiodarone forms colloidal states in aqueous solutions and forms low-molecular-weight aggregates that precipitate through dilution^34^. Therefore, the more pronounced visual changes observed in RM than in SM may be due to amiodarone aggregation, which is influenced by pH and the different mixing conditions of SM and RM. Particle size distribution assessments indicated that in SM, the particle size gradually increased from 7.8 nm to 81.3 nm as the furosemide dose increased. In contrast, in RM, a large particle size of 200 nm was observed at the lowest furosemide dose of 5 mg/h, indicating a notable difference in particle size distribution between the two mixing methods. The flow within infusion lines does not strictly conform to either laminar or turbulent regimes but rather exhibits complex transitional behavior^24^. Such flow complexity may induce localized shear forces, non-uniform mixing zones, and fluctuating drug concentrations, which may facilitate colloid destabilization and aggregation. The dynamic mixing conditions simulated by RM likely reflected these real-world flow complexities, contributing to the differences in particle size distribution and appearance compared with the static SM condition. Furthermore, mixing in two rather than one stage results in the formation of larger aggregates when a flocculant is added to a colloidal suspension^35,36^. In the one-stage method, the particle surface is covered by a flocculant, thereby inhibiting aggregation. In contrast, unreacted particles remained in the two-stage method, allowing for secondary aggregation and formation of larger aggregates. The RM method may promote aggregation in a manner similar to the two-step mixing process, due to the sequential mixing environment, where initially, mixing occurs at the branching sites, followed by continued mixing along the infusion route. Based on these results, the differences observed in the incompatibility between SM and RM were attributed to the distinct aggregation behavior of amiodarone under the two mixing conditions. In other words, the compatibility between amiodarone and furosemide is influenced by pH-induced changes in the solubility and aggregation behavior under different mixing conditions.

This study had some limitations. First, compatibility evaluations were conducted after a relatively short mixing period of 15 min, which may not have fully captured the long-term stability changes that occur during continuous infusion. Second, temporary administration interruptions and the diverse designs of Y-site connectors used in clinical practice may result in interactions that differ from those observed under the controlled experimental conditions of this study. Third, our study has limitations related to formulation excipients. The precise identity and concentration of the pH regulator in the furosemide formulation were not disclosed by the manufacturer, and this agent likely influenced the final pH and compatibility outcomes. In addition, the 5% dextrose solution used as a vehicle has an inherently acidic pH (typically ranging from 3.5 to 6.5), which may also affect the final pH of the mixture. These uncharacterized factors should be considered when interpreting our results and warrant further investigation in future studies. Furthermore, the RM method may be considered a form of flow reaction chemistry, but since our study relied on offline measurements, it may not have fully captured real-time precipitation dynamics. Future online monitoring might better clarify flow-dependent incompatibility mechanisms. Finally, as this study focused on a single drug combination, the applicability of these findings to other commonly used ICU drug combinations remains unclear.

## Methods

### Sample preparation

Mixed amiodarone and furosemide samples were prepared using two methods to simulate continuous infusion in the ICU. The first is the SM method, which is a widely used approach in compatibility testing. In this method, amiodarone and furosemide solutions are mixed directly in screw-cap tubes at the clinical administration ratio (Fig. 1a). The second method is the RM method, which utilizes an infusion line and syringe pumps to closely replicate the clinical setting of continuous infusion. Specifically, an experimental system was constructed to simulate clinical administration conditions, including the infusion rate and mixing environment, using standard clinical infusion equipment (Fig. 1b).

Amiodarone is commonly administered by diluting 75 mg/1.5 mL of the drug with 48.5 mL of 5% dextrose (Otsuka Pharmaceutical Co., Ltd., Tokyo, Japan) to prepare a 1.5 mg/mL solution^37^, followed by continuous infusion at a maintenance rate of 25.5 mg/h (17 mL/h) (Sanofi Co., Ltd., Tokyo, Japan; Interview Form “Ancaron^®^,” revised July 2024). Furosemide was used undiluted at a concentration of 10 mg/mL (Lasix^®^ 100 mg/10 mL; Nichi-Iko Pharmaceutical Co., Ltd., Tokyo, Japan), with dosing rates of 5, 8, and 10 mg/h selected within the clinical range (Table 1)^38,39^. Based on these protocols, for the SM method, amiodarone at 1.5 mg/mL (1.70 mL, equivalent to 25.5 mg/h) was mixed with furosemide at 10 mg/mL (50, 80, or 100 µL, corresponding to 5, 8, or 10 mg/h, respectively) in a glass screw-cap tube (No. 3, capacity: 9 mL; Maruemu Co., Ltd., Tokyo, Japan) and stirred (Table 2). For the RM method, syringes (50 and 10 mL; Terumo Co., Ltd., Tokyo, Japan) filled with 1.5 mg/mL amiodarone and 10 mg/mL furosemide were connected to a syringe pump (FP-1000; Melquest Co., Ltd., Toyama, Japan). Amiodarone was infused at 25.5 mg/h, and furosemide was infused at 5, 8, or 10 mg/h through a three-way stopcock (Table 2).

**Table 1:** Medications used for compatibility testing.

Drugs	Amiodarone (Ancaron^®^)	Furosemide (Lasix^®^)
Strength	150 mg/3 mL	100 mg/10 mL
Additives	Benzyl alcohol Polysorbate 80	Sodium chloride pH regulator
pH	3.5–4.5	8.6–9.6
Manufacturer	SANOFI	Nichi-Iko Pharmaceutical
Lot	1K0820A 2K085A 3K086A	1K207A 3K220A

**Table 2:** Setting of mixing volumes and infusion rates for Amiodarone and Furosemide in simulation and route mixing methods.

Drugs	Amiodarone	Furosemide		
Tested concentration	1.5 mg/mL	10 mg/mL		
SM				
Mixed volume	1.70 mL	50.0 µL	80.0 µL	100 µL
RM				
Mixed dose	25.5 mg/h	5.0 mg/h	8.0 mg/h	10.0 mg/h
(Mixed volume)	(17 mL/h)	(0.5 mL/h)	(0.8 mL/h)	(1 mL/h)

Samples were prepared in triplicate using the SM and RM methods to obtain three independent samples for each condition. All samples were prepared at room temperature (20–25 °C). Notably, amiodarone hydrochloride adsorbs onto polyvinyl chloride (PVC) infusion sets, and leaching of the plasticizer di(2-ethylhexyl) phthalate (DEHP) from DEHP-containing PVC infusion sets is a concern. Therefore, infusion routes made of PVC- and DEHP-free materials were used in the RM method. Specifically, a three-way stopcock extension tube (TS-WR1735L; Terumo Co., Ltd.) with an internal diameter of 2.1 mm, a length of 50 cm, and a volume of 1.7 mL was connected to an extension tube (SA-ET211100; Terumo Co., Ltd.) with an internal diameter of 2.1 mm, a length of 20 cm, and a volume of 0.8 mL.

### Evaluation of visual changes

All mixed samples were collected in glass screw-cap tubes and macroscopically evaluated by the same investigator under standard laboratory conditions using a black background and fluorescent light. The evaluation criteria included color change, precipitate formation, and turbidity. Changes in color (420 nm) and turbidity (550 nm) were quantitatively assessed using a microplate reader (SH-1000Lab; CORONA ELECTRIC Co., Ltd., Niigata, Japan)^40^. Changes in absorbance were calculated relative to the absorbance of the sample prepared using the SM method with amiodarone and furosemide at 5 mg/h, which showed no apparent visual changes.

### Powder X-ray diffraction analysis

PXRD analysis was performed to investigate the composition of the white precipitates generated by the SM and RM methods. For crystallinity evaluation, a turbid mixture was prepared using a furosemide infusion rate of 20 mg/h, which produced more pronounced turbidity and a higher precipitate recovery rate than 10 mg/h under RM. The resulting turbid mixture was allowed to settle, and the precipitate was dried and analyzed using PXRD (MiniFlex 600 X-ray diffractometer; Rigaku Co., Ltd., Tokyo, Japan). Measurement conditions were as follows: Cu-Kα radiation as the source, tube voltage of 30 kV, tube current of 15 mA, scanning range of 5.0–35.0°, and scan speed of 10°/min. The composition of the white precipitate was determined by comparing the diffraction patterns with those of each drug alone. The PXRD patterns of furosemide were obtained from previously reported polymorphs, form I (No. 1161545) and form II (No. 761770), retrieved from the Cambridge Crystallographic Data Center. The PXRD pattern of amiodarone was obtained from a standard amiodarone hydrochloride sample (FUJIFILM WAKO PURE CHEMICAL Co., Ltd., Osaka, Japan).

### pH measurement

Changes in the pH of mixed drug solutions can significantly affect their stability^41^. The pH of each solution was measured using a pH meter (F-2000 PI; HORIBA Co., Ltd., Kyoto, Japan). Measurements were performed for each sample, and the obtained pH values are presented as the mean ± standard deviation.

### Particle size distribution measurement

The particle size distribution of both the SM and RM samples was measured immediately after preparation to assess the particle size of the turbid mixed solutions resulting from formulation changes. DLS was used for these measurements. The particle size was measured using a Nanoparsec SZ-100V2 series instrument (HORIBA Co., Ltd., Kyoto, Japan) under the following conditions: wavelength, 532 nm; temperature, 25 °C; viscosity, 0.898 cP; and refractive index, 1.333. Measurements were conducted on undiluted samples, and each sample was analyzed in triplicate. The mean values were used to construct the graphs.

### Statistical analyses

Statistical analyses were performed using JMP^®^ version 8 (SAS Institute, Cary, NC, USA). One-way analysis of variance was performed, followed by Tukey’s multiple comparison test. Data are presented as the mean ± standard deviation or standard error, and p-values < 0.05 were considered significant.

## Conclusion

This study demonstrates that the mixing conditions significantly influence the compatibility of amiodarone and furosemide. Notably, the RM method, which simulates clinical intravenous infusion routes, resulted in increased turbidity and particle growth compared to conventional static mixing. These findings suggest that traditional in vitro compatibility tests may underestimate the risk of precipitation and aggregation during intravenous administration. In high-risk settings such as the ICU, dynamic simulation methods such as RM may be more appropriate for assessing the potential for catheter occlusion and drug delivery failure. Based on our findings, no visible changes were observed at lower infusion rates of furosemide, although alterations in particle size were detected. The clinical significance of these changes for pharmacological activity and pharmacokinetics remains uncertain and warrants further investigation. Therefore, co-administration of amiodarone and furosemide through the same intravenous line should be avoided, and separate administration routes are recommended. This study provides novel evidence supporting the need to re-evaluate the standard compatibility testing methods commonly used in clinical practice. However, further studies are warranted to confirm these findings under extended infusion times, across diverse administration routes, and under biologically relevant conditions, including the presence of serum proteins. Such efforts are essential to generalize the current findings and facilitate their translation into clinical practice.

## Data Availability

The data used to support the findings of this study are available from the corresponding author upon request.
